# Organic–mineral fertilization modulates microbial communities and nutrient-cycling genes in saline–alkali soil

**DOI:** 10.3389/fmicb.2026.1776848

**Published:** 2026-03-16

**Authors:** Haonan Chen, Manli Duan, Quanjiu Wang, Beibei Zhou, Rupan Yan, Xiaopeng Chen, Mingjiang Deng

**Affiliations:** 1 State Key Laboratory of Water Engineering Ecology and Environment in Arid Area, Xi’an University of Technology, Xi’an, China; 2 Xinjiang Future Irrigation District Engineering Technology Research Center, Urumchi, China

**Keywords:** C, N, and P cycle genes, functional genes, organic fertilizer, saline–alkali soil, soil microbial communities

## Abstract

**Introduction:**

Soil salinization constrains crop production in arid regions, yet the microbial and functional mechanisms underlying organic–mineral co-application in saline–alkali soils remain unclear.

**Methods:**

A pot experiment with sorghum–sudangrass was conducted in a saline–alkali soil under five fertilization regimes with equal total N but different proportions of organic N. Soil physicochemical properties were measured at the seedling and maturity stages, and rhizosphere bacterial communities and C, N and P cycling genes at maturity were characterized by 16S rRNA gene sequencing and SmartChip high-throughput qPCR.

**Results:**

Organic–mineral fertilization decreased soil pH and total salt content and increased soil organic matter, total N and available P relative to mineral fertilizer alone, with the strongest improvements under the 50% organic–50% mineral N regime. Organic inputs increased bacterial Shannon diversity and evenness and shifted community composition, enriching *Actinobacteriota*, *Firmicutes*, *Bacillus* and *Pseudarthrobacter*. The balanced regime increased genes involved in C degradation/fixation, N fixation and P mineralization/polyphosphate metabolism (e.g., *xylA, acsA, mct, nifH, phoD, ppx*), whereas mineral-only fertilization favored nitrification/denitrification and methane oxidation genes (e.g., *amoA2, nirK, nirS, pmoA*), indicating a higher potential for N losses.

**Discussion:**

Multivariate analyses identified soil pH, total salt, organic matter and total N as primary regulators of bacterial communities and functional gene profiles. Moderate organic–mineral co-application, particularly the 50%–50% regime, improves soil conditions and strengthens nutrient-cycling potential in saline–alkali sorghum–sudangrass systems.

## 1 Introduction

Saline–alkali soils, characterized by high salt contents, elevated pH, and high sodium ion concentrations, are widely distributed throughout the world (Ivushkin et al., 2019). Excessive salinity disrupts soil structure and markedly reduces water infiltration, permeability, and soil porosity (Zahedifar, 2020), thereby impeding water movement and aeration. It also hampers the buildup and persistence of organic matter in soil and limits nutrient availability, ultimately resulting in reduced soil fertility (Tian et al., 2023). Under saline–alkali conditions, soil microbial activity and enzyme reaction rates are strongly inhibited, weakening nutrient cycling and exerting negative effects on crop growth (Xu et al., 2020). Consequently, soil salinization has become a major limitation to crop production and is increasingly recognized as an important driver of worldwide food insecurity. Improving the physicochemical conditions and nutrient availability of saline–alkali soils is therefore essential to restore their long-term productivity. In practice, a range of management options has been adopted, including the use of chemical and biological amendments, installation of drainage systems and plant-based reclamation measures (Ma et al., 2011). These measures are widely used because they can improve soil structure, increase the availability of nutrients, and promote microbial activities. In particular, organic fertilizers are highly beneficial for improving the fertility of saline–alkali soils.

Organic fertilizers are environmentally friendly inputs with vital importance for agricultural ecosystems and global environmental change (Sayer and Cassman, 2013; Seufert et al., 2012). Many investigations have demonstrated that when organic amendments are used together with mineral fertilizers, soil physical quality and chemical fertility are enhanced and nutrient cycling is intensified. At the same time, this practice reorganizes soil microbial community structure and alters its functional attributes (Chen et al., 2020; Sun et al., 2021; Wang et al., 2021). Organic fertilizer application is widely regarded as an effective option for improving saline–alkali soils. It promotes the enrichment of copiotrophic bacterial groups, including Proteobacteria and Actinobacteriota, and increases bacterial *α*-diversity by modifying soil pH and organic matter levels. Functional genes within these microbial communities, particularly those involved in nutrient cycling, show pronounced positive responses to organic inputs. Organic fertilization leads to higher copy numbers of genes associated with C, N, and P transformations, such as the carbon fixation gene *acsA*, the nitrogen fixation marker *nifH* and the phosphonate mineralization gene *phnK*. Meta-analyses of field experiments have indicated that organic fertilizer application markedly stimulates functional genes associated with nitrification and denitrification, and that its stimulatory effects on N-cycling genes are greater than those of mineral fertilizer alone (Xu et al., 2023). Khan et al. (2023) further demonstrated that organic fertilization enhanced the activity of P-transforming enzymes and promoted the enrichment of the phosphatase genes *phoC* and *phoD*, which are central to the hydrolytic mobilization of organic P (Po) and thus to global phosphorus cycling. By contrast, mineral fertilization markedly enriches functional genes associated with methane oxidation, nitrogen degradation, nitrification and anaerobic ammonium oxidation. When manure is applied together with mineral fertilizers, denitrification is further stimulated, which can lead to increased N₂O emissions (Hu et al., 2022). Different fertilization regimes can modify soil physicochemical conditions and thereby indirectly affect the cycling of carbon, nitrogen and phosphorus. Earlier work has shown that soil pH, total nitrogen (TN) and organic matter (OM) are positively associated with these biogeochemical processes (Mitsuta et al., 2025; Pan et al., 2025). In addition, Li et al. (2020) identified soil pH, TN and nitrate nitrogen (NO₃^−^-N) as key drivers of the size and structure of N cycling microbial communities in agricultural soils. However, in saline–alkali soil systems, the interactions between soil properties and nutrient-cycling functional genes under organic fertilization remain poorly understood.

Therefore, optimizing the rational application of organic fertilizer to improve the plant growth environment and soil productivity in saline–alkali soils has become a critical issue. In light of earlier work on how organic fertilization alters soil physicochemical conditions and nutrient cycling, we tested the following hypotheses in this study: (1) the addition of organic fertilizer alters nutrient contents in saline–alkali soils, thereby increasing soil bacterial diversity and modifying community composition; (2) appropriate inputs of organic fertilizer reduce soil pH and enhance nutrient availability, thereby increasing the abundance and functional potential of functional genes mediating carbon, nitrogen and phosphorus transformations in saline–alkali soils; (3) organic fertilizer application is correlated with soil properties and indirectly affects nutrient-cycling genes by modifying the structure of the bacterial community.

## 2 Materials and methods

### 2.1 Experimental design and treatments

In this pot experiment, urea (CH₄N₂O, 46.7% N) and monopotassium phosphate (KH₂PO₄, 28.7% K and 22.8% P) were applied as mineral fertilizers. The organic fertilizer was an aerobic compost prepared from cattle manure and wheat straw. Saline–alkali soil was sampled from the 0–20 cm layer of cropland in the Hotan Demonstration Area, Xinjiang, China, and classified as a saline–alkali loam with 1.81% clay, 8.09% silt and 90.35% sand, with a total salt content (TS) of 1.73 g kg^−1^ (Table 1).

**Table 1A:** Basic physicochemical properties of saline soil.

Properties	pH	TS (g/kg)	Organic matter (g/kg)	Available phosphorus (mg/kg)	Available potassium (g/kg)	Soil bulk density (g/cm^3^)	Initial mass moisture content (%)
Saline–alkali soil	8.76	1.73	2.06	34.62	1.18	1.55	5

**Table 1B:** Basic physicochemical properties of organic fertilizer.

Properties	TOC (g/kg)	TN (g/kg)	NO_3_^–^-N (mg/kg)	NH_4_^+^-N (mg/kg)	C/N	pH	Initial mass moisture content (%)
Organic fertilizer	238	13.5	62.8	10.1	19.5	7.71	7.52

The pot trial was carried out in a greenhouse at Xi’an University of Technology (Shaanxi, China) from 26 August to 27 December 2022. Cylindrical PVC tubes (18 cm in diameter, 50 cm in height) served as pots, and the initial bulk density of the soil was 1.60 g/cm^3^. The chemical fertilizer application rates were 642 kg/ha for urea (CH_4_N_2_O) and 439 kg/ha for KH_2_PO_4_. Prior to the pot trial, the saline–alkali soil was pre-incubated for 2 weeks at 25 °C in the dark to stimulate microbial activity, and soil water content was kept at 60% of field capacity throughout this period. Nitrogen in the organic and chemical fertilizers was applied in different proportions and thoroughly mixed with the soil according to the following treatments: T1, 100% N from organic fertilizer; T2, 70% N from organic fertilizer + 30% N from chemical fertilizer; T3, 50% N from organic fertilizer + 50% N from chemical fertilizer; T4, 30% N from organic fertilizer + 70% N from chemical fertilizer; and T5, 100% N from chemical fertilizer. All treatments were arranged in triplicate. After fertilization and pre-sowing irrigation, half of the total chemical fertilizer was applied as a basal dressing, and the remaining half was applied equally at the tillering and jointing stages. Each pot received 15 kg of soil, and 10 sorghum–Sudan grass seeds were sown per pot. At the seedling stage, five plants with good growth were selected from each pot. Irrigation was applied as needed to keep soil water content at approximately 70% of field capacity during the trial.

### 2.2 Sample collection and analysis

During sorghum–Sudan grass growth in 2022, rhizosphere soil was sampled with a soil auger at the seedling (19 September) and maturity (27 December) stages. After collection, each soil sample was split into two subsamples. One subsample was kept at 4 °C for subsequent physicochemical analyses. The remaining subsample was freeze-dried (Songyuan, Beijing, China), ground in a cryogenic mill (Retsch Z200, Germany), sieved through a 0.5 mm mesh and immediately frozen at −80 °C for downstream molecular analyses. The basic chemical properties of the saline–alkali soil were determined according to the methods outlined in “*Agrochemical Analysis of Saline–Alkali Soils*.” Soil pH and electrical conductivity (EC) were determined on 1:5 (w/v) soil–water suspensions using a pH meter and a conductivity meter, respectively (Mettler Toledo, Switzerland). Ammonium and nitrate nitrogen (NH₄^+^–N, NO₃^−^–N) were extracted with 1 mol L^−1^ KCl and quantified with a fully automated discrete analyzer (SmartChem 450, AMS Alliance, France). Available P was obtained by extraction with 0.5 mol L^−1^ NaHCO₃ and was also measured on the SmartChem 450. Available K was extracted with 1 mol L^−1^ neutral ammonium acetate and determined by atomic absorption spectrophotometry. Total N was analyzed by the Kjeldahl digestion procedure, and soil organic C was measured using the potassium dichromate oxidation with external heating.

### 2.3 Microbial diversity analysis

DNA for 16S rRNA sequencing was extracted from rhizosphere soil collected at the maturity stage. Soil DNA was extracted from 0.5 g of freeze-dried soil samples using the FastDNA kit (MP Biomedicals, USA) following the manufacturer’s instructions. DNA concentration and purity were determined with an Epoch micro-volume spectrophotometer (BioTek, USA), and qualified DNA samples were stored at −20 °C until analysis. The V4 region of the bacterial 16S rRNA gene was amplified with primers 515F (5′-GTGCCAGCMGCCGCGGTAA-3′) and 806R (5′-GGACTACHVGGGTWTCTAAT-3′), and amplicons were sequenced on the Ion S5 XL platform (Thermo Fisher Scientific, USA).

Raw reads were processed with the UPARSE pipeline for quality filtering, and chimeric sequences were removed using USEARCH. High-quality sequences were clustered into operational taxonomic units (OTUs) at 97% similarity, and an OTU table was constructed and rarefied to a uniform sequencing depth before downstream analyses. Based on the rarefied OTU table, bacterial *α*-diversity indices (Chao1 richness, Shannon diversity, Simpson diversity and Pielou’s evenness) and Bray–Curtis dissimilarities were calculated to characterize community richness, diversity and compositional differences among fertilization treatments. Principal coordinates analysis (PCoA) was used to visualize patterns in bacterial community structure across treatments, and heatmaps were generated to illustrate the distribution of dominant OTUs. Differences among treatments were evaluated using the statistical procedures described in the “Statistical analysis” section.

### 2.4 C, N, and P functional gene quantification

Primers specific to key genes involved in C, N, and P cycling were pre-loaded on a high-throughput SmartChip qPCR array (Magigene Biotechnology Co., Ltd., Guangzhou, China). Functional genes were quantified in parallel using a SmartChip real-time PCR system (Magigene, Guangzhou, China) with primers pre-loaded on a high-throughput qPCR array. Total microbial DNA extracted from soil samples as described in Section 2.3 was used as the template, and DNA concentration and purity were checked prior to analysis. Qualified DNA samples were submitted to Magigene, where an automated liquid-handling system dispensed reaction mixtures from sample and primer plates into the wells of the SmartChip qPCR array. High-throughput qPCR amplification and fluorescence detection were then performed on the SmartChip real-time PCR system.

Cycle threshold (Ct) values for functional genes associated with C, N, and P cycling were obtained from the SmartChip qPCR analysis software and exported as a matrix for downstream analysis. Standard curves generated from serial dilutions of reference templates were used to convert Ct values into gene copy numbers for each target. Gene copy numbers were then normalized within each sample to obtain the relative abundances of individual C, N, and P cycling functional genes, which were used to construct functional gene profiles for subsequent multivariate and univariate analyses. The primer sequences and functional gene categories related to C, N, and P cycling are listed in Supplementary Table S1 (Zheng et al., 2018).

### 2.5 Statistical analysis

All statistical analyses were conducted using IBM SPSS Statistics 26 (SPSS Inc., Chicago, USA) and R version 4.3.1. Data were checked for normality and homogeneity of variance before analysis. Variables meeting these assumptions were analyzed by one-way analysis of variance (ANOVA) followed by Tukey’s *post hoc* test, with *p* < 0.05 considered statistically significant; for non-normal or heteroscedastic data, the Kruskal–Wallis test was applied. Results are reported as mean ± standard deviation (SD), with three replicates per treatment (*n* = 3).

Bray–Curtis dissimilarities derived from the rarefied OTU table (Section 2.3) and from C, N, and P cycling functional gene profiles (Section 2.4) were used for multivariate analyses. Principal coordinates analysis (PCoA) based on Bray–Curtis dissimilarities was used to visualize differences in bacterial community composition and functional gene profiles among fertilization treatments. Permutational multivariate analysis of variance (PERMANOVA; adonis function in the “vegan” package, 999 permutations) was applied to test for overall treatment effects on bacterial communities and on C, N, and P cycling functional gene profiles, and the associated R^2^ and *p* values were reported as the proportion of variance explained by fertilization. Relative abundance data (bacterial taxa at phylum and genus levels, and C, N, and P related functional genes) were arcsine square-root transformed prior to ANOVA to better satisfy model assumptions, and treatment effects on these variables were then evaluated by one-way ANOVA with Tukey’s multiple comparisons.

Relationships between soil physicochemical properties and functional gene relative abundances were examined using Mantel tests based on distance matrices of environmental variables and gene profiles (vegan, 999 permutations). Random forest regression models were fitted with the “randomForest” package, and variable importance was assessed using “rfPermute” to identify key environmental predictors of C, N, and P cycling functional genes. Partial least squares path modeling (PLS-PM) was performed with the “plspm” package to quantify direct and indirect effects of fertilization regime and soil properties on bacterial diversity and functional genes, using path coefficients, coefficients of determination (R^2^) and total effects to interpret the models.

## 3 Results

### 3.1 Changes in physicochemical properties of saline soil under varying organic fertilizer application rates

**Figure 1:**
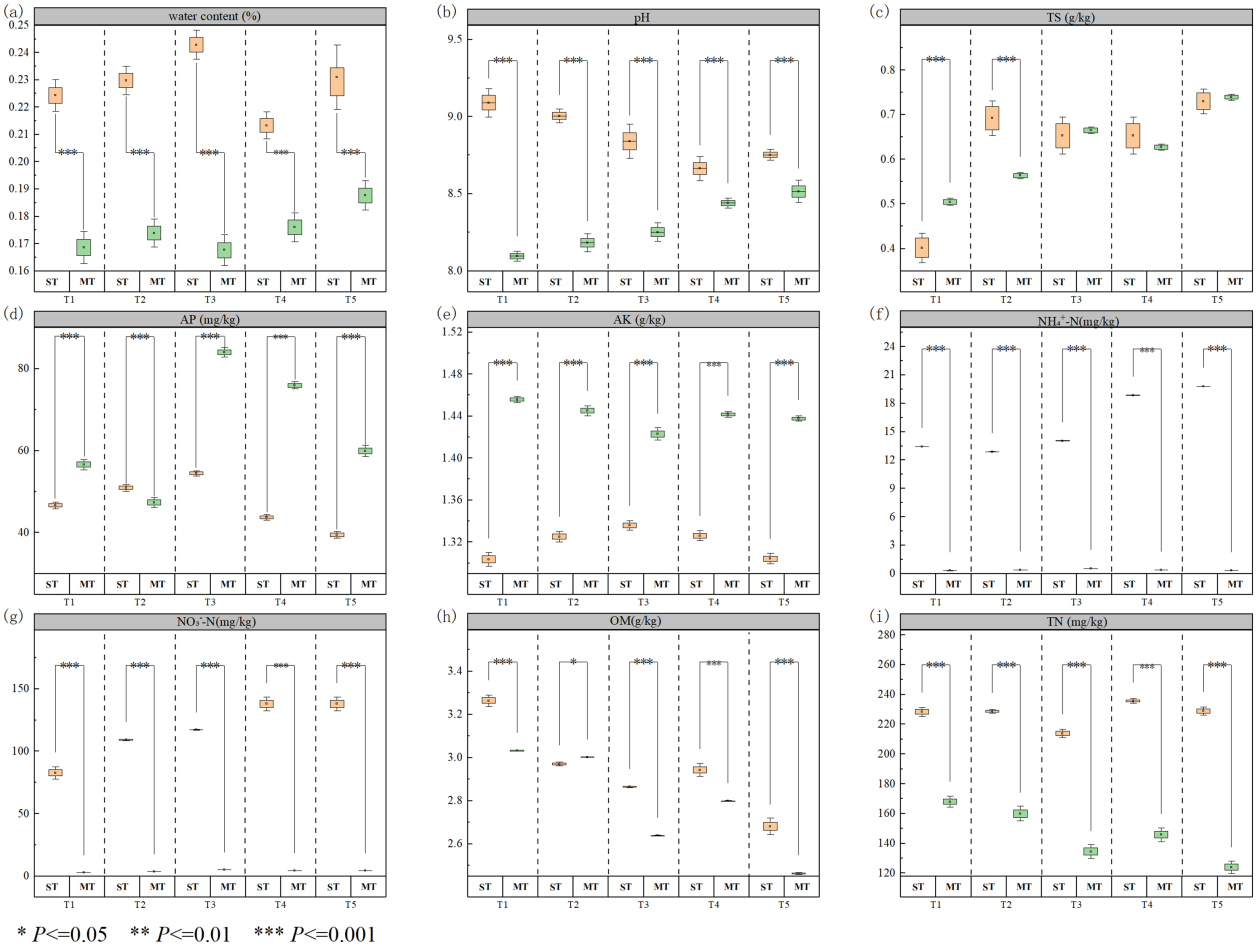
Effects of different organic–mineral fertilizer ratios on saline–alkali soil properties at the seedling stage (ST) and maturity stage (MT): **(a)** water content (%), **(b)** pH, **(c)** total salt content (TS; g kg^−1^), **(d)** available phosphorus (AP; mg kg^−1^), **(e)** available potassium (AK; g kg^−1^), **(f)** ammonium nitrogen (NH₄^+^-N; mg kg^−1^), **(g)** nitrate nitrogen (NO₃^−^-N; mg kg^−1^), **(h)** organic matter (OM; g kg^−1^), and **(i)** total nitrogen (TN; mg kg^−1^). Values are presented as mean ± SD (*n* = 3). Asterisks indicate significant differences between ST and MT within the same treatment based on one-way ANOVA (*p < 0.05, **p < 0.01, ***p < 0.001).

The effects of the fertilization treatments on soil pH, moisture, total salt content, and nutrient status at the seedling and maturity stages are shown in Figure 1. From the seedling to the maturity stage, soil pH decreased under all treatments, with the largest reduction in T1 (100% organic fertilizer, 11.2%) and the smallest in T5 (100% mineral fertilizer, 2.3%). For soil moisture and salinity, T1, T2, and T3 generally maintained higher water contents than the other treatments, and at maturity the soil moisture in T3 was 9.7 and 5.8% higher than in T1 and T2, respectively. Total salt content at maturity increased most strongly in T1 (24.4%), whereas the increase in T5 was minimal (1.2%), with T2–T4 showing intermediate levels.

Regarding nutrient supply, the increase in available P from seedling to maturity was significantly greater in T3 (54.2%) than in the other treatments (20.7% in T1, 51.8% in T5; *p* < 0.05). Available K rose most in T1 (11.7%), exceeding that in all other treatments. For inorganic N forms, soil NH₄^+^-N declined sharply in all treatments, with the greatest reduction in T5 (98.4%), followed by T3 (96.1%). At maturity, the NO₃^−^-N and total N (TN) contents in T3 reached 5.29 mg/kg and 134.6 mg/kg, respectively. Soil organic matter (OM) in T3 decreased by 6.9% from the seedling to the maturity stage. However, its OM content at maturity (2.64 g/kg) remained higher than that in T5 (2.46 g/kg) (Figure 1).

**Figure 2:**
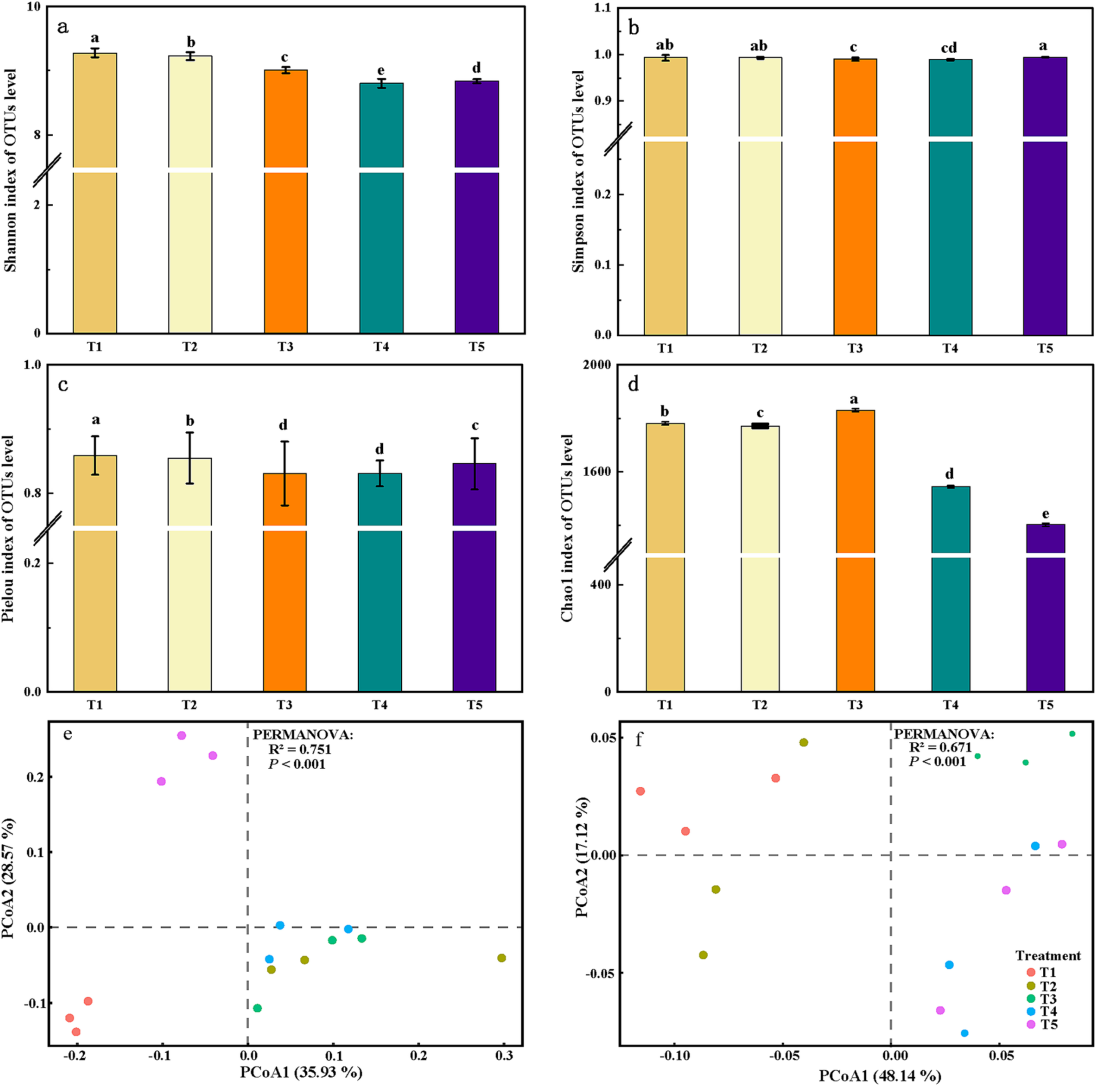
The *α*-diversity and *β*-diversity of soil bacterial communities and functional genes under different fertilization regimes. **(a)** Shannon, **(b)** Simpson, **(c)** Pielou’s evenness, and **(d)** Chao1 richness indices of soil bacterial communities under different organic–mineral fertilization regimes. Principal coordinates analysis (PCoA) based on Bray–Curtis dissimilarities showing **(e)** bacterial community composition and **(f)** C, N, and P cycling functional genes under different fertilization regimes. R^2^ and *p* values in panels **(e)** and **(f)** are derived from PERMANOVA and proportion of variation in bacterial communities and functional gene profiles explained by the fertilization regimes. Different lowercase letters in panels **(a–d)** indicate significant differences among treatments (*p* < 0.05).

### 3.2 Effects of organic–mineral fertilization on soil bacterial diversity and community composition

#### 3.2.1 Bacterial community diversity

Bacterial *α*-diversity declined significantly with decreasing organic fertilizer proportion (*p* < 0.05) (Figure 2). T1 had the highest Pielou’s evenness and Shannon indices, indicating the most even bacterial community among the treatments. In contrast, the Chao1 index reached its maximum in T3, being 2.68% higher than in T1, whereas T5 had the lowest Chao1, reflecting the lowest species richness.

The *β*-diversity of bacterial communities and functional genes was also strongly affected by the fertilization regimes. PCoA based on Bray–Curtis dissimilarities showed tight clustering of replicates within each treatment and clear separation among treatments; T1 and T2 grouped closely together, while the mineral-N-dominated treatments T3–T5 were clearly distant from them. PERMANOVA revealed that the fertilization regimes explained 75.1% of the variation in bacterial community structure and 67.1% of the variation in functional gene composition (both *p* < 0.001), indicating that the ratio of organic to mineral fertilizer is a major driver of microbial community structure and nutrient-cycling functions in saline–alkali soil.

#### 3.2.2 Bacterial community structure

**Figure 3:**
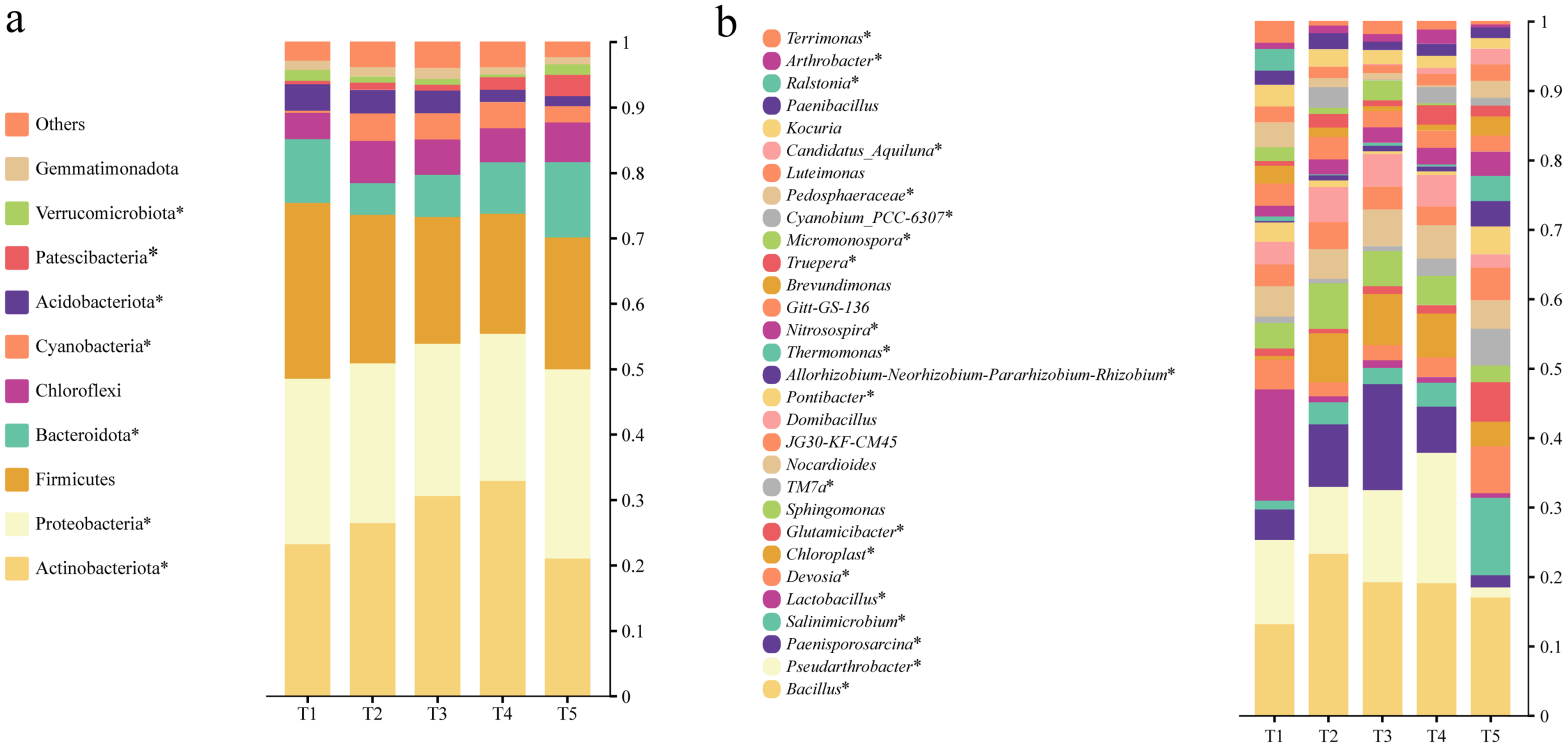
Relative abundances of bacterial communities at the phylum **(a)** and genus **(b)** levels in saline–alkali soil under different organic fertilizer application rates (T1–T5). Asterisks next to taxon names indicate taxa with significantly different relative abundances among fertilization treatments (*p* < 0.05; one-way ANOVA on arcsine square-root–transformed data followed by Tukey’s *post hoc* test).

As shown in Figure 3a, Actinobacteriota, Proteobacteria and Firmicutes dominated the bacterial communities in the saline–alkali soil samples, with relative abundances of 20.95–32.87%, 22.46–28.95% and 18.42–26.90%, respectively, across the different treatments. Organic–mineral fertilization regimes significantly modified the distribution of these major phyla. Actinobacteriota was particularly enriched in T3 and T4, where its relative abundance was 24.5–56.9% greater than in T1 and T5. In contrast, T1 contained the highest proportion of Firmicutes, exceeding the other treatments by 18.5–46.1%.

Genus-level analysis (Figure 3b) showed that *Bacillus*, *Pseudarthrobacter* and *Paenisporosarcina* were the dominant genera, together accounting for 36.8% of the total relative abundance. Overall, treatment T3 (50% organic fertilizer) had the highest total relative abundance of these dominant genera (47.07%), with most of them being more abundant than in T1 (100% organic fertilizer) and T5 (100% mineral fertilizer). In T3, the relative abundance of *Bacillus* was 45.99% greater than in T1 and 13.18% greater than in T5; however, T2 (70% organic fertilizer) showed the highest *Bacillus* abundance, which was a further 20.89% higher than in T3. For *Pseudarthrobacter* and *Paenisporosarcina*, their abundances in T3 were 9.1% and 2.49-fold higher than in T1, and approximately 8-fold and 7.71-fold higher than in T5, respectively.

### 3.3 Changes in soil functional gene abundances with increasing organic fertilizer inputs

**Figure 4:**
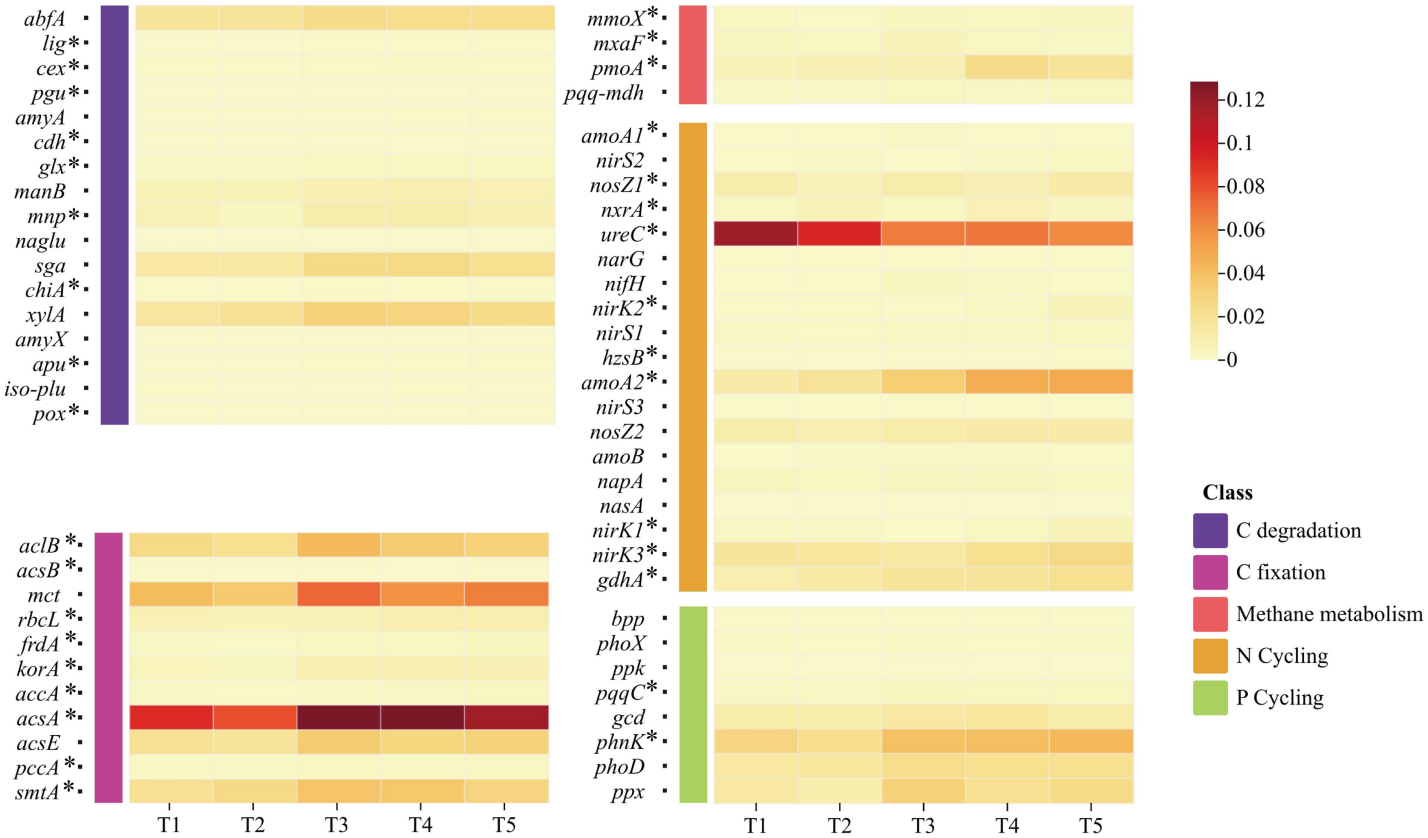
Heatmaps showing the abundances of functional genes involved in carbon (C), nitrogen (N), and phosphorus (P) cycling in saline–alkali soil under different organic fertilizer application treatments (T1–T5). Asterisks next to gene names indicate genes with significantly different abundances among fertilization treatments (*p* < 0.05; one-way ANOVA on arcsine square-root–transformed data followed by Tukey’s post hoc test).

**Figure 5:**
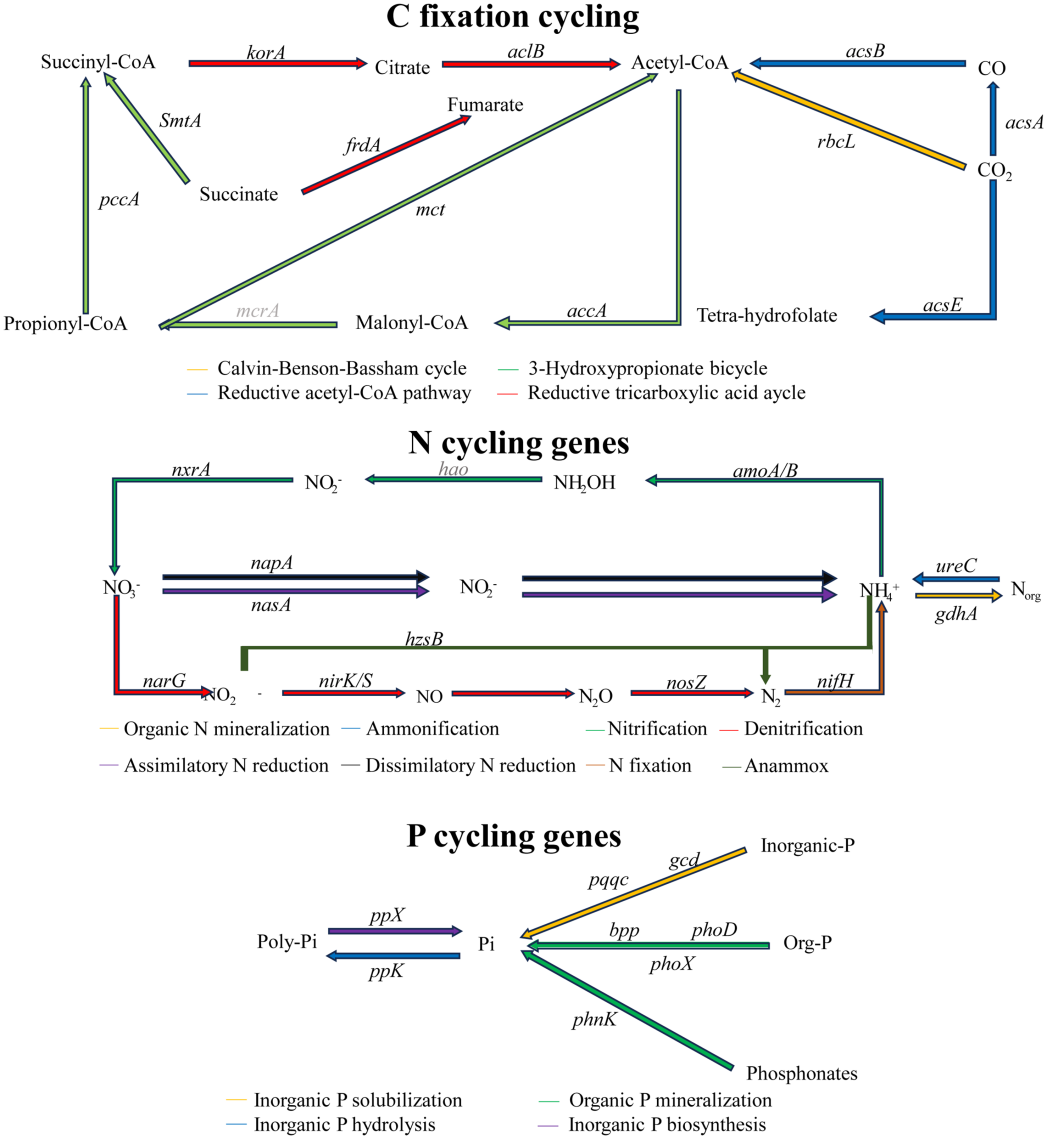
Schematic overview of key processes and representative functional genes involved in C fixation, N cycling and P cycling in saline–alkali soil.

A total of 63 functional genes related to carbon (C), nitrogen (N), and phosphorus (P) transformations were detected in the Hotan saline–alkali soil (Figure 4). Among them, N-cycling genes (32 genes) were the most abundant, followed by C-cycling genes (19 genes, including C degradation and C fixation), whereas P-cycling genes were relatively fewer. According to the pathway diagram, these genes correspond to organic C decomposition, methane oxidation, and CO₂ assimilation/fixation in the C cycle; to organic N mineralization and ammonification, nitrification, nitrate reduction and denitrification, and N fixation in the N cycle; and to organic P mineralization, phosphonate utilization, and polyphosphate turnover in the P cycle, and the abundance of multiple functional genes differed significantly among fertilization treatments (Figure 5).

Within C cycling, the hemicellulose-degradation gene *xylA* involved in labile organic C decomposition showed the highest abundance in T3, being 92.6 and 26.1% higher than in T1 and T5, respectively; the lignin-degradation gene *mnp* involved in recalcitrant C decomposition peaked in T5, increasing by 34.5% relative to T1. During CO₂ assimilation/fixation, the gene *acsA* participating in the reductive acetyl-CoA pathway and the gene *mct* participating in the 3-hydroxypropionate (3-HP) bicycle reached their highest abundances in T3; *acsA* was 39.7% higher than in T1, and *mct* was 75.49 and 29.47% higher than in T1 and T5, respectively; the gene *pmoA* participating in methane oxidation was highest in T5, being 199.1% higher than in T1.

For the N cycle, the gene *amoA2* participating in the initial step of nitrification was most enriched in T5 (100% mineral fertilizer), increasing by 270.6% relative to T1 (100% organic fertilizer); the gene *ureC* participating in ammonification showed the highest abundance in T1, being 90.6% higher than in T5. The gene *narG* participating in nitrate reduction was 61.6% higher in T5 than in T1, and the genes *nosZ1* and *nosZ2* participating in the terminal N₂O reduction step of denitrification were relatively high in both T1 and T5, with *nosZ1* in T5 being 25.3% higher than in T1; the gene *nifH* participating in N fixation showed the highest abundance in T3, being 208.6% higher than in T1.

In P cycling, the gene *phnK* participating in phosphonate utilization and the gene *phoD* participating in organic P mineralization increased under T5, being 46.7 and 46.4% higher than in T1, respectively; under T3, the gene *phoD* participating in organic P mineralization and the gene *ppx* participating in polyphosphate turnover reached their highest abundances, being 60.1 and 106.7% higher than in T1, respectively. Overall, organic–inorganic fertilization (T3) and sole inorganic fertilization (T5) exhibited differentiated stimulation patterns on C-, N-, and P-transformation genes.

### 3.4 Linkages of microbial functional genes with soil properties

**Figure 6:**
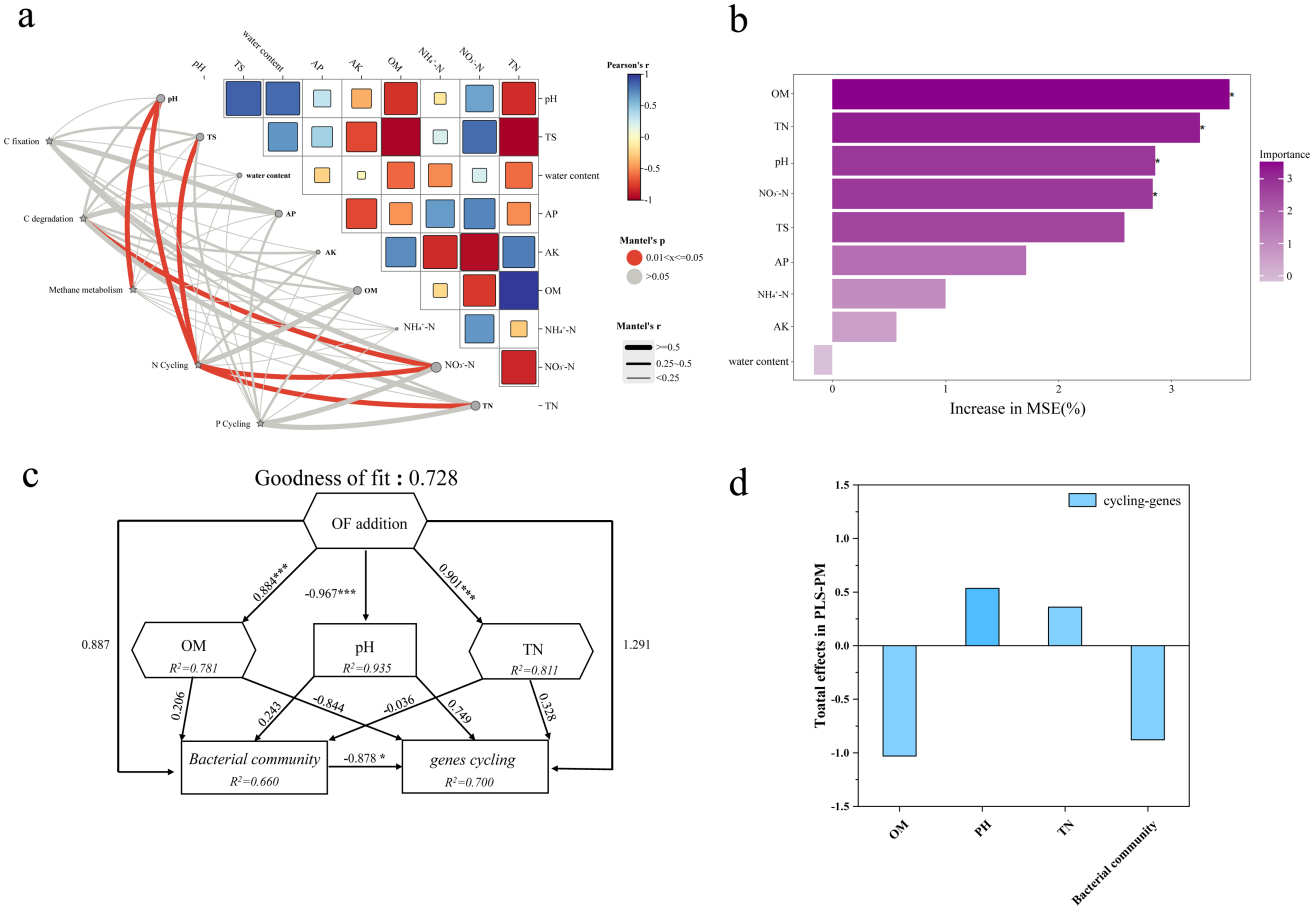
Environmental drivers of carbon (C), nitrogen (N), and phosphorus (P) cycling genes. **(a)** Partial Mantel tests were used to assess correlations between functional genes and environmental factors (gray and red lines represent non-significant (*p* > 0.05) and significant (0.01 < *p* < 0.05) correlations, respectively). Pairwise comparisons of environmental factors (Pearson’s correlation coefficients) are shown in the upper triangle. Color gradients represent the strengths of correlations. Edge widths correspond to the Mantel statistic, and edge colors indicate significance based on 999 permutations. **(b)** Random forest analysis identified the most important predictors of functional gene abundances, where the importance of each factor was evaluated based on the increase in the mean squared error (MSE). Asterisks indicate statistically significant predictors (* *p* < 0.05, ** *p* < 0.01). **(c)** Partial least squares-path modeling (PLS-PM) revealed the relationships among organic fertilizer addition, soil properties, bacterial diversity, and abundance of C, N, and P cycling genes. Path coefficients near arrows indicate the magnitudes of direct effects between variables. GOF indicates the model’s goodness-of-fit. R^2^ values above each response variable represent the proportion of variance explained. **(d)** The panel summarizes the standardized total effects (direct + indirect effects) derived from PLS-PM. Asterisks indicate significant effects (* *p* < 0.05; ** *p* < 0.01; *** *p* < 0.001).

**Figure 7:**
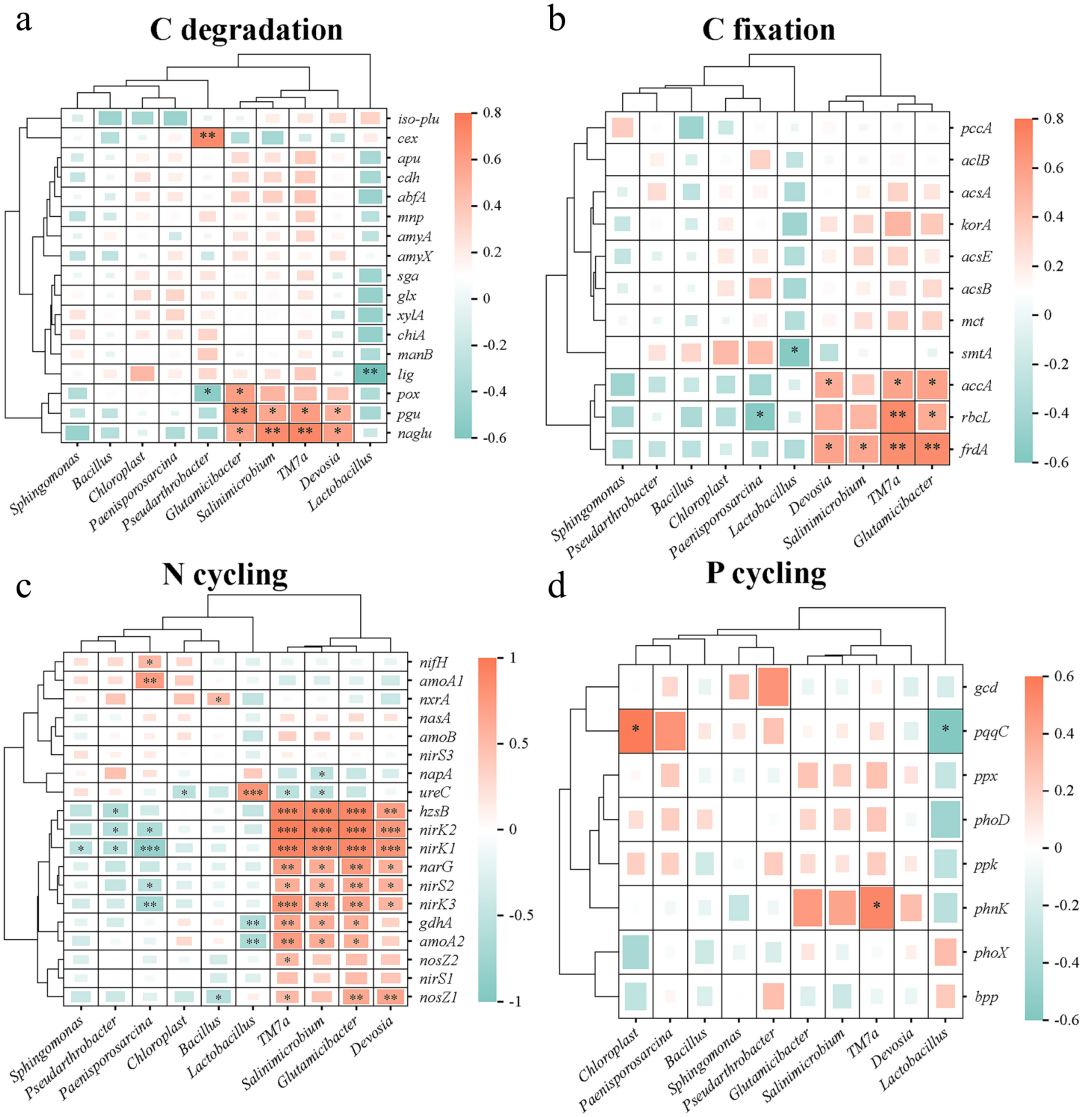
Pearson correlation heatmaps between dominant bacterial genera and functional genes involved in C degradation, C fixation, N cycling and P cycling. **(a)** C degradation; **(b)** C fixation; **(c)** N cycling; **(d)** P cycling. Colors indicate correlation coefficients (red, positive; blue-green, negative; scale shows r values). Dendrograms represent hierarchical clustering. Significance: * *p* < 0.05, ** *p* < 0.01, *** *p* < 0.001.

Figure 6 summarizes the relationships between functional genes mediating soil carbon (C), nitrogen (N) and phosphorus (P) transformations and key environmental variables. Mantel tests showed significant relationships of N and P cycle genes with pH, total salt content (TS), NO₃^−^-N and TN (*p* < 0.05), whereas C cycle genes were not significantly related to these physicochemical properties (Figure 6a). Random forest analysis identified OM, TN and pH as the main soil factors driving variation in functional gene abundances, followed by NO₃^−^-N and TS (Figure 6b). The PLS-PM model exhibited an acceptable overall fit (GOF = 0.728), explaining 78.1–93.5% of the variation in major soil properties, 66.0% of the variation in the bacterial community, and 70.0% of the variation in functional genes (Figure 6c). Organic fertilizer (OF) addition significantly increased OM and TN and decreased pH (path coefficients 0.884***, 0.901*** and −0.967***, respectively), and exerted an overall positive indirect effect on C, N, and P cycling functional genes by altering soil properties and bacterial community composition (Figures 6c,d). On this basis, we further applied Pearson correlation analysis to pair dominant genera with key nutrient-cycling genes (*p* < 0.05). The results showed that *TM7a* was significantly and positively correlated with the C-degradation gene *naglu*, the C-fixation gene *rbcL*, the key N-cycling gene *nirK2*, and the P-cycling gene *phnK*; meanwhile, *Pseudarthrobacter* was also significantly and positively correlated with the C-degradation gene *cex*. These results together indicate that fertilization influences the responses of nutrient-cycling functional genes by altering soil properties and reshaping key bacterial taxa (Figures 7a–d).

## 4 Discussion

### 4.1 Soil microbial community responses to varying fertilization regimes

Organic fertilizer application substantially enhanced bacterial *α*-diversity in the saline–alkali soil and caused marked shifts in community composition and structure, thereby supporting our first hypothesis that organic fertilization increases bacterial species richness and evenness and reshapes microbial community structure. In this study, all treatments receiving organic inputs (T1–T3) showed significantly higher α-diversity than the treatment with mineral fertilizer alone (T5). Among them, T1 exhibited the highest Pielou’s evenness and Shannon index, whereas T3 had the highest Chao1 index, indicating that an appropriate combination of organic and mineral fertilizers not only helps maintain high community evenness but also promotes species richness. This pattern agrees with earlier findings that organic fertilizer application promotes higher microbial diversity (Yang et al., 2025). Improvement in soil physicochemical properties is likely one of the main mechanisms underlying this increase in diversity. The incorporation of organic fertilizer into saline–alkali soil can reduce salinization and increase organic matter and nutrient supply, thereby providing a more favorable habitat for microorganisms (Hou et al., 2025). The PCoA based on Bray–Curtis dissimilarities further showed that different fertilization regimes strongly drive the differentiation of bacterial communities and C, N, and P cycling functional gene profiles, suggesting that a balanced supply of organic and mineral nutrients both satisfies microbial C and N demands and alleviates the salt and nutrient stress caused by excessive mineral fertilizers, thus maintaining higher community diversity and strengthening nutrient-cycling functions (Cruz-Paredes et al., 2021).

In addition to *α*-diversity, organic fertilization also markedly altered the composition of dominant taxa. In this study, Actinobacteriota and Proteobacteria were the dominant phyla in saline–alkali soil, and their relative abundances, particularly that of Actinobacteriota, were significantly increased under T3 and T4. One plausible explanation is that co-application of organic and mineral fertilizers supplies abundant organic substrates and creates a more favorable nutrient environment for these groups (Zhang et al., 2025). Organic inputs supply more labile organic C and N, thereby favoring copiotrophic groups such as Actinobacteriota and Proteobacteria, which are efficient decomposers of soil organic C pools and are strongly associated with soil fertility (Duan et al., 2025). By contrast, T1 (100% organic fertilizer) showed the highest abundance of Firmicutes, indicating that the high-organic-matter environment created by sole organic fertilization favored this phylum, whose members are often efficient degraders of complex organic substrates (Francioli et al., 2016; Tu et al., 2018; Xun et al., 2016). At the genus level, *Bacillus* was more abundant in T2 (70% organic fertilizer) and T3 (50% organic fertilizer) than in T1 (100% organic fertilizer) and T5 (100% mineral fertilizer), indicating that intermediate organic–mineral ratios favor the enrichment of this beneficial genus, consistent with previous reports (Liu et al., 2021; Du et al., 2022). Compared with 100% organic fertilization, *Bacillus* was significantly enriched under T3, whereas its abundance was strongly suppressed under T5, implying that sole mineral fertilization may lead to insufficient available C sources and local nutrient imbalances, thereby constraining the growth of beneficial bacteria (Cui et al., 2022).

Overall, an appropriate combination of organic and mineral fertilizers not only enhances bacterial diversity but also promotes the enrichment of potentially beneficial genera such as *Bacillus*, *Pseudarthrobacter* and *Paenisporosarcina*, which is conducive to improving soil health in saline–alkali systems. These patterns are consistent with our first hypothesis that organic fertilizer inputs increase bacterial diversity and restructure community composition in saline–alkali soil.

### 4.2 Effects of organic fertilizer addition on nutrient cycling genes

Functional microorganisms drive core soil processes such as organic matter mineralization, biological nitrogen fixation, and contaminant transformation by harboring key genes involved in multiple element cycles (Hu et al., 2024; Keshri et al., 2013). The efficiency of these microbially mediated nutrient cycles is regulated by the dynamic balance between microbial metabolic demands and environmental nutrient supply, in which soil bacteria govern nutrient turnover pathways through extracellular enzyme release and the regulation of gene expression (Hu et al., 2022). Previous studies have shown that organic fertilizer inputs can significantly increase the abundance of functional genes related to nutrient cycling, such as the cellulose-degradation gene *celA*, the ammonia monooxygenase gene *amoA*, and the dissimilatory sulfite reductase gene *dsrA* (Ren et al., 2025), providing a theoretical basis for directionally regulating soil microbial functions through fertilization strategies. For the results of this study, T3 (organic–mineral co-application) mainly enhanced two key links in the C cycle, namely the decomposition of labile organic carbon and CO₂ assimilation/fixation, improved the N-fixation function in the N cycle, and promoted the processes of organic P mineralization, phosphonate utilization, and polyphosphate turnover in the P cycle, thereby overall strengthening the microbial metabolic potential for C, N, and P.

Our results indicate that, in the Hotan saline–alkali soil, variations in the abundance of C-degradation functional genes were closely associated with the mode of N fertilizer co-application. Labile soil C pools are crucial for soil fertility, productivity, and biodiversity (Lal, 2004; Lal, 2016). In this study, hemicellulose-degradation–related genes (e.g., *abfA*, *xylA*, and *manB*) were overall higher under T3, suggesting that moderate organic–mineral co-application may preferentially promote the turnover of labile organic C pools rather than simply enhancing the decomposition of more recalcitrant C, which is consistent with the notion that labile components are activated first (Yuan et al., 2025). Organic fertilizer inputs supply exogenous C and increase soil organic matter levels (Huan et al., 2014); on this basis, genes involved in CO₂ assimilation/fixation in T3—such as *acsA* participating in the reductive acetyl-CoA pathway and *mct* participating in the 3-hydroxypropionate (3-HP) cycle—were relatively more abundant (Coskun et al., 2016), indicating that co-application may enhance the potential bacterial capacity for CO₂ fixation and contribute to the re-accumulation of soil organic C (Guo et al., 2019). In addition, compared with the methylotrophy/methanol-utilization–related genes *pqq-mdh* and *mxaF*, the methane-oxidation genes *pmoA* and *mmoX* showed higher abundances under T5, suggesting that under high mineral N input, saline–alkali soils may exhibit a stronger potential for CH₄ oxidation, thereby helping to suppress the risk of CH₄ emissions to some extent (Fry et al., 2023).

Soil N cycling is driven by diverse microorganisms, including diazotrophs, ammonifiers, nitrifiers, and denitrifiers (Levy-Booth et al., 2014). In this study, the ammonification gene *ureC*, as a dominant gene in the N cycle, peaked under T1 (100% organic fertilizer), indicating that organic fertilization strengthened the tendency for N to be converted to NH₄^+^, thereby promoting the transformation of organic N into inorganic N and favoring N supply. The N-fixation gene *nifH* was most abundant under T3, suggesting that the synergy of a moderate mineral N input with organic fertilizer may provide a more balanced C and N resource supply and a more suitable microenvironment for diazotrophs, thereby enhancing the biological N-fixation potential of saline–alkali soil and benefiting crop nutrient acquisition. In contrast, *amoA2* was significantly enriched under T5 (100% mineral fertilizer), indicating that sole mineral fertilization may markedly enhance nitrification potential, which could in turn lead to NO₃^−^-N accumulation and increase the risk of N loss via leaching and runoff (Hooper and Terry, 1979). The denitrification functional genes *nirS3* and *nasA* showed the highest relative abundances under T3. As a nitrite reductase gene, *nirS3* drives the further reduction of nitrite to nitric oxide (NO), representing a key step linked to gaseous emissions during denitrification in saline–alkali soils (Tang et al., 2022). Although denitrification genes were active under T3, this treatment may have alleviated gaseous N losses by reducing transient nitrite accumulation via the slow-release nature of organic N. By comparison, T5 (100% chemical fertilizer) showed overall dominance of denitrification-related genes such as *nirS2*, *nirK1*, *nirK2*, and *narG*, suggesting a higher likelihood of N₂O emissions under T5. Excess N accumulated under T5 may be partially lost through gaseous pathways, which is consistent with previous reports (Bouwman et al., 2013). Therefore, combined organic–mineral fertilization may reduce the likelihood of N losses in saline–alkali soils.

In the P cycle, *phoD* (alkaline phosphatase) can represent the potential for organic P mineralization, *phnK* represents phosphonate utilization, *ppx* (together with related genes such as *ppk*) reflects polyphosphate (polyP) turnover, and *gcd* is often associated with inorganic phosphate solubilization and organic acid–mediated activation processes (Enebe and Babalola, 2021; Srivastava et al., 2022). Hu et al. (2023) pointed out that manure inputs not only provide available P but also stimulate microbial P-solubilizing and organic P mineralization processes, thereby expanding the pool of plant-available P. It should be emphasized that, under the high-pH conditions of saline–alkali soils, even when mineral P is applied, microbe- and plant-available P may still be constrained due to precipitation/fixation. Therefore, the increases in *phnK* and *phoD* under T5 may be explained by the fact that, in the saline–alkali context, microorganisms may respond to limited available P or localized rhizosphere P acquisition pressure by enhancing the expression of genes related to phosphonate utilization and organic P mineralization. In contrast, *phoD* and *ppx* remained at relatively high levels under T3 (and in some cases T4), indicating that moderate organic–mineral co-application may, through the synergistic supply of C sources and mineral nutrients and by improving the rhizosphere microenvironment, be more favorable for the proliferation of microbial groups harboring *phoD* and polyP-turnover–related genes, thereby enhancing the potential for organic P mineralization and polyP turnover (Ragot et al., 2015; Sakurai et al., 2008; Bi et al., 2020; Fraser et al., 2015; Hu et al., 2018). In addition, the increases in *gcd* and *ppx* observed under T3/T4 are consistent with previous observations (Zhang et al., 2023; Liu et al., 2024), suggesting that under some fertilization regimes, the activation of sparingly soluble inorganic P and microbial polyP turnover may jointly constitute an important pathway supplying plant-available P. Overall, sole application of organic or mineral fertilizers may, in some cases, reduce the relative abundances of these key genes (Chen et al., 2017), whereas co-application is more likely to maintain the integrated potential of key nodes related to P acquisition and turnover.

### 4.3 Relationships between functional genes and environmental variables

Mantel tests in this study showed that several environmental factors, including pH, total salt content (TS), TN, NO₃^−^-N and OM, were significantly correlated with a subset of nutrient-cycling functional genes, with the strongest relationships observed for N and P cycling genes, whereas C cycling genes showed generally weaker overall correlations (Figure 6a). This indicates, on the one hand, that soil physicochemical properties are important drivers of the spatial distribution of functional genes, and, on the other hand, that different C cycling genes do not respond to fertilization and environmental gradients in a consistent manner and are more strongly governed by the quality of organic substrates and enzyme-mediated processes. As a result, C cycling genes tend to exhibit weaker aggregate correlations at the whole-system scale (Liu et al., 2010; Shu et al., 2021). Random forest analysis further showed that OM, TN and pH exerted the strongest influence on functional gene abundances, with NO₃^−^-N and TS being of secondary importance (Figure 6b). On this basis, the PLS-PM model provided an integrated view of the relationships among organic fertilizer (OF) addition, soil properties, bacterial communities and functional genes: OF addition exerted indirect effects on functional gene abundances by markedly increasing OM and TN, lowering pH, and altering bacterial community structure (Ren et al., 2025). To further refine the relationship between the bacterial community and functional genes, we applied Pearson correlation analysis (*p* < 0.05) to pair dominant genera with key nutrient-cycling genes (Figures 7a–d): *TM7a* was significantly and positively correlated with *naglu*, *rbcL*, *nirK2* and *phnK*, and *Pseudarthrobacter* was also significantly and positively correlated with *cex*. The genus-level correlation patterns between taxa and functional genes are consistent with the PLS-PM results showing that community structure drives functional gene expression. This indicates that fertilization-induced microbial community reassembly may further lead to corresponding changes in the potential of key nutrient-cycling functions in soil.

In saline–alkali soils, pH and salinity are key factors constraining microbial activity and nutrient cycling (Wang et al., 2017). Considering the observed changes in soil physicochemical properties (Figure 1) together with the path coefficients in the PLS-PM model, it is evident that organic fertilizer application reduced soil pH, adjusted TS levels, and increased OM and TN, thereby helping to alleviate Na^+^ toxicity and salt stress and improve microbial habitats. Previous studies have reported that fertilization-induced shifts in pH can directly regulate the expression of C, N, and P cycling functional genes (Li et al., 2020). The significant associations between N- and P-cycle genes and pH, TS, TN and NO₃^−^-N observed in this study suggest that, under alleviated alkalinity and salinity stress, these two gene groups are particularly sensitive to improvements in soil conditions.

OM and TN jointly shape the functional gene network from the perspective of C and N supply. First, organic fertilization markedly increased OM (Figure 1), adding exogenous labile C sources and a fraction of organic N, which provided sufficient energy and ecological niches for microorganisms carrying C and P cycling genes, thereby promoting the expression and diversity of these genes (Malik et al., 2018). Second, elevated TN reshaped the partitioning of NH₄^+^-N and NO₃^−^-N and altered C–N coupling relationships, thereby influencing organic matter decomposition rates and shifting the relative importance of individual N transformation pathways (Long et al., 2012; Neff et al., 2002). At the same time, changes in N: P stoichiometry feed back to regulate the expression of P cycling genes such as *phoD*, *ppx* and *phnK*, thereby influencing inorganic P solubilization and organic P mineralization processes (Risch et al., 2019; Yang et al., 2021).

Collectively, key environmental factors such as pH, TS, OM and TN jointly shape the soil microbial community and its functional gene composition, thereby exerting pronounced environmental filtering and metabolic regulation on C, N, and P nutrient-cycling processes in saline–alkali soils. The synchronous changes between dominant genera and nutrient-cycling genes further identify candidate taxa that co-vary with key cycling genes. In addition, considering the significant regulatory effects of OF addition on pH, OM and TN, it can be inferred that an appropriate organic–mineral fertilization regime can be directly associated with nutrient-cycling genes through environmental conditions, and can also indirectly drive functional gene responses by altering community structure.

## 5 Conclusion

From a practical perspective, we recommend the 50% organic N + 50% mineral N treatment (T3) as the most applicable fertilization regime for sorghum–sudangrass production in saline–alkaline soils. Overall, this study clarifies how organic–mineral co-application reshapes soil microbial communities and enhances nutrient-cycling potential in saline–alkali soils. Compared with mineral fertilization alone, T3 provided a more balanced C and N supply and alleviated salt stress, which increased bacterial diversity and shifted community composition (e.g., enrichment of Actinobacteriota and *Bacillus*). This treatment also enriched key genes involved in C fixation (*acsA*, *mct*), N fixation (*nifH*) and P turnover (*phoD*, *ppx*), consistent with improved soil organic matter accumulation and nutrient availability. Collectively, these findings support our hypotheses that organic–mineral co-application enhances microbial diversity and nutrient-cycling functions and links soil properties with microbially mediated processes in saline–alkali soil.

The environmental driver model showed that organic fertilizer inputs reshaped microbial community structure and functional gene networks by lowering soil pH and salt stress and increasing OM content, with soil pH, OM and TN emerging as key determinants of the expression patterns of genes associated with C, N, and P transformations. Overall, the combined organic–mineral fertilization strategy improved both soil physicochemical properties and microbial metabolic functions, thereby achieving the dual goals of desalinization and salt control and efficient nutrient use in saline–alkali soil, and providing an optimized, microbially mediated pathway for the amelioration of salinized soils in arid regions. As this was a greenhouse pot experiment, field-scale validation across saline–alkaline gradients is needed; nevertheless, our results provide a mechanistic basis for designing fertilization regimes aimed at sustaining sorghum–sudangrass production while rehabilitating saline–alkali soils.

## Data Availability

The raw 16S rRNA amplicon sequencing reads generated in this study have been deposited in the NCBI Sequence Read Archive (SRA) under BioProject accession PRJNA1428215.
